# The significance of connectedness: avoidant personality disorder patients’ subjective experiences of change after attending a specialized treatment program

**DOI:** 10.3389/fpsyg.2024.1412665

**Published:** 2024-07-08

**Authors:** Kristine Dahl, Kjetil Bremer, Theresa Wilberg

**Affiliations:** ^1^Group Therapy Team, Aust-Agder County Outpatient Psychiatric Unit, Sorlandet Hospital, Kristiansand, Norway; ^2^Norwegian National Advisory Unit on Personality Psychiatry (NAPP), Oslo University Hospital, Oslo, Norway; ^3^Division of Mental Health and Addiction, Department of Research and Innovation, Oslo University Hospital, Oslo, Norway; ^4^Institute of Clinical Medicine, University of Oslo, Oslo, Norway

**Keywords:** avoidant personality disorder, specialized treatment, psychotherapy, subjective experience, qualitative research

## Abstract

**Objectives:**

This study aimed to inquire into the subjective experiences and meaning-making of change of people diagnosed with avoidant personality disorder (AvPD) after attending a treatment program developed for AvPD.

**Methods:**

Eighteen AvPD patients were interviewed 1 year after completing their treatment using a semi-structured interview guide. The interviews were analyzed through reflexive thematic analysis.

**Results:**

Three main themes were found to capture the various subjective experiences of change. The first main theme “being more alive” included the subthemes “talking and listening together” and “opening up and grounding into myself.” The second main theme was “still longing for more,” and the third main theme “I cannot even manage therapy” included the subthemes “as if we were together” and “capitulation.”

**Conclusion:**

Although these findings may not be specific to AvPD, they shed light on the importance of attending to the dynamic interplay of intersubjectivity, social motivations, and agency in a therapeutic context. Discovering a sense of agency within an interpersonal context in which the patient feels connected may lead to them opening up for development in accordance with their social motivational intentions.

## 1 Introduction

What does it mean for people diagnosed with avoidant personality disorder (AvPD) to work for change in a specialized form of therapy that seeks to adapt to their specific challenges? Psychotherapy can be viewed as a co-constructive process that takes place within the matrix of interpersonal relations in a group setting or within a dyadic therapeutic relationship, which are influenced by the unique characteristics and meaning-making of the persons present and the dynamic between them (Clarkin, 2012; Shean, 2013; Timulak and Keogh, 2017). In this co-constructive process, the clients’ active engagement is considered pivotal for therapeutic change (Acke et al., 2022). People diagnosed with AvPD have been found to seek therapy to find greater self-confidence and inner strength to better stand their ground and cope with adversities as well as to get to know themselves better and feel connected and free to do what they want without fearing others’ evaluations and reactions (Sorensen et al., 2019b). At the same time, like in all relational settings, the psychological challenges associated with AvPD will naturally play out in therapy. Therefore, AvPD therapy may require adaptions to facilitate the development of the therapeutic relationship and/or group cohesion as well as specific therapeutic techniques to resolve or decrease patients’ challenges and symptoms.

Avoidant personality disorder is characterized by “a pervasive pattern of social inhibition, feelings of inadequacy, and hypersensitivity to negative evaluation, beginning by early adulthood and present in a variety of contexts” and concerns difficulties related to fear of rejection and criticism as well as feelings of inferiority and restraint in intimate relations due to fear of being shamed or ridiculed (American Psychiatric Association, 1994, p. 672). Recent research has contributed to a richer understanding of the disorder beyond the diagnostic criteria. People diagnosed with AvPD are found to struggle with their sense of self and identity, impaired emotional awareness, problems with monitoring one’s own and others internal states, and impaired reflective functioning (Nicolo et al., 2011; Normann-Eide et al., 2015; Antonsen et al., 2016; Moroni et al., 2016; Johansen et al., 2018; Sorensen et al., 2019a). Avoidant personality disorder is associated with significant interpersonal and attachment difficulties, and patients often present extensive social impairment (Beeney et al., 2015; Eikenaes et al., 2015; Kvarstein et al., 2021). Many AvPD patients describe developmental experiences characterized by emotional distance and inhibition, a sense of detached insecurity in close relationships, reduced verbal sharing of psychological states, and deficient social learning experiences (Sorensen et al., 2020). People diagnosed with AvPD may struggle with ongoing insecurity and doubt, both longing for and fearing connecting relationally to others as well as to themselves, thereby resorting to various coping strategies that could alienate them from their sense of self and agency (Millon, 1992; Sorensen et al., 2019a; Weme et al., 2023).

From a therapist’s perspective, the aforementioned characteristics present significant challenges to establishing and maintaining a treatment alliance that may help AvPD patients overcome their fearfulness and relational ambivalence in order to be able to make use of the therapeutic techniques and relationships (with therapists or group members) offered in therapy (Pettersen, 2021). However, therapists lack knowledge of patients’ own subjective experience of their treatment and change. In Sorensen et al.’s (2019b) qualitative study, some of the participants diagnosed with AvPD conveyed that they felt a sense of being managed in therapy. They hoped for some explanations and direction for change, but as their therapy progressed, some described a sense of not making themselves understood or not feeling understood, being told what to do, and becoming increasingly inactive and discontent. Those who recounted positive change underscored the importance of a genuine, emotionally accessible, warm, and active therapist as well as a sense of increasing vitality, engagement, and emerging trust. These findings underscore the importance of interpersonal connection and collaboration in therapy as well as how the interpersonal problems that characterize AvPD may lead to subservience and pseudo-alliance. However, it is worth noting that these descriptions are from the participants’ experiences with therapy and change in general (Sorensen et al., 2019b) and not from more newly developed specialized treatments/procedures for AvPD (e.g., Baljé et al., 2016; Dimaggio et al., 2017; Simonsen et al., 2019; Bachrach and Arntz, 2021; Simonsen et al., 2022; Wilberg et al., 2023).

It is important to note that although existing psychotherapy studies achieve significant relief for many AvPD patients, research also shows varying outcomes and even little or no progression for some with a more severe pathology, despite them receiving long-term psychotherapy (Kvarstein and Karterud, 2012). The potential of getting worse in therapy is generally present for psychotherapy patients (e.g., Curran et al., 2019; Strauss, 2021; Woodbridge et al., 2022), and this is probably also the case for people diagnosed with AvPD. Studies on the effectiveness of psychotherapy are important to evaluate whether positive changes likely correlate with the therapy in question. Yet to date, these studies do not inform us of how or why a positive change, no change, or a negative change took place. Therefore, patients’ own sense-making of change may shed a light on the implications of quantitative findings and, as such, give direction to further treatment development and research.

The aim of the present study was to inquire into the subjective experiences and sense-making of change of AvPD patients after attending a treatment program developed for AvPD. To our knowledge, this is the first qualitative study to explore how people diagnosed with AvPD might experience change after participating in therapy specifically focused on their psychological challenges. Gaining a better understanding of patients’ sense-making of therapy processes is of particular importance as more specialized treatments are developed and studied.

## 2 Materials and methods

### 2.1 Procedures

#### 2.1.1 Setting and design

This study was part of a small-scale pilot study of a treatment for patients with AvPD conducted from 2012 to 2019. The study took place at Oslo University Hospital’s Outpatient Clinic for Specialized Treatment of Personality Disorders.

The psychotherapy was inspired by mentalization-based therapy (MBT) (Bateman and Fonagy, 2016), and metacognitive interpersonal therapy (MIT) (Dimaggio et al., 2015) The treatment modality was a combination of weekly group and individual therapy. Therapists were guided by the MIT perspectives on motivational systems, maladaptive interpersonal schemas and an emphasis on positive affects. However, they mainly applied central MBT principles in the group therapy, such as focusing on the patients’ mental states, challenging patients to self-reflect when exploring concrete episodes from their current lives, adapting a therapist not-knowing stance, and focusing on affects and relations. The main purpose of the individual therapy was to support the patients’ participation in the group therapy through preparing themes and interpersonal episodes and practicing social sharing.

Each group included eight to nine patients. Initially, the treatment was a time-limited one-year program with a closed group format. However, the clinical impression and preliminary data from the first two patient groups indicated that the treatment length was too short. Consequently, the treatment was extended to a two-year program, and after 6 months of treatment, the patients in the second group were offered the choice of continuing the treatment for a maximum of 2 years. Thus, the treatment changed to a slow open program, i.e., when the enrolled patients ended their treatment, new patients were admitted to the group. The group therapy included psychotherapeutic work and psychoeducation on various topics considered relevant. The weekly 45-min individual sessions were primarily aimed at supporting the patients’ participation in the group therapy, but was also a valuable opportunity to share personal topics they were not able to discuss in the group. More detailed information on the pilot project, as well as a more thorough description of the assessment, theoretical orientations and the group and individual therapy structure, is provided in Wilberg et al.’s (2023) work.

Information on the pilot study and the possibility of referring patients to the program were published on the clinic’s website and sent to outpatient psychiatric clinics in the region. Patients were referred from outpatient psychiatric clinics, by private practitioners with contracts with the regional health authorities, or by general practitioners. Upon referral, the patients completed extensive diagnostic and clinical evaluation, including various self-report questionnaires, several of which were repeated during and following the treatment.

Two separate qualitative interviews were conducted during the follow-up period. The first was conducted 3 months after the treatment ended, and the second, which provided data for the current study, was conducted 1 year after the treatment was planned to end. This means that patients in the one-year program were interviewed 2 years after the start of their treatment, whereas patients in the two-year program were interviewed 3 years after the start of their treatment. This one-year follow-up interview focused on the patients’ subjective experiences of change and their reflections on what might have contributed to the changes or lack thereof.

### 2.2 Participants

#### 2.2.1 Interviewees

The inclusion criteria for the pilot study were a diagnosis of AvPD and motivation for change and treatment focusing on interpersonal exposure inside and outside the treatment setting. To ensure that the patients had some arena for social exposure, the patients were required to have a minimum of social contact outside the family, be in some kind of work or study context, or have realistic plans for such activities. Initially, patients aged between 20 and 45 years old were eligible for inclusion in the study. However, the participant age range was later limited to between 20 and 30 years.

Exclusion criteria were co-occurring schizotypal, schizoid, paranoid, or antisocial personality disorder; current alcohol or substance dependence; psychotic disorders; bipolar I disorder; severe PTSD; untreated ADHD (adult form); pervasive developmental disorder (e.g., Asperger’s syndrome); organic syndromes or any other disorder that entails total withdrawal and isolation; homelessness; and insufficient fluency in Norwegian language. Patients were enrolled in the study during the period from 2012 to 2016, and the one-year follow-up investigation ended in 2019.

Twenty-eight patients were initially included in the pilot study. However, of the 10 patients offered 1 year of treatment, one was diagnosed with Asperger’s syndrome during treatment and consequently excluded from further analyses. Twenty patients were available for the one-year follow-up, but two of them were excluded from the present analyses due to their development of other severe psychiatric conditions during and following the treatment. Thus, the present study includes 18 patients: five from the one-year treatment program and 13 from the two-year treatment program.

The sample comprised 11 females and seven males with a median age of 26 years (range = 20–35). At the start of the treatment, 17 patients were diagnosed with AvPD according to the DSM-IV (American Psychiatric Association, 1994), assessed with SCID II (First et al., 1997). One patient had subthreshold AvPD fulfilling three AvPD criteria and was diagnosed as a personality disorder not otherwise specified. All the patients had only one personality disorder diagnosis, except one patient with co-occurring borderline personality disorder. The sample was characterized by high levels of interpersonal problems and symptom distress and moderate to severe social dysfunction (Table 1). Only one patient lived in a romantic relationship and many had many previous treatments. The median treatment length in the present study was 15 months (range = 6–27) months.

**Table 1:** Levels of symptom distress, interpersonal problems, and psychosocial functioning at the beginning of treatment (*N* = 18).

	Mean (SD)
Symptom distress, GSI^a^	1.62 (0.49)
Interpersonal problems, CIP^b^	1.80 (0.49)
Work and social adjustment, WSAS^c^	26.61 (6.10)
Psychosocial functioning, GAF^d^	52.44 (3.84)

^a^ Symptom Checklist-90-Revised, Global Severity Index (Derogatis, 1994). Based on a Norwegian sample a score of 0.80 is regarded as cutoff for non-clinical values (Pedersen and Karterud, 2004).

^b^ Circumplex of Interpersonal Problems (Pedersen, 2002). A value of 0.85 is regarded as a clinical/non-clinical cut-score.

^c^ Work and Social Adjustment Scale (Mundt et al., 2002). Total scores above 30 denote severe disability, scores between 15 and 30 denote moderate impairment, and scores below 15 can be regarded as mild impairment or disability.

^d^ Global Assessment of Functioning Scale (American Psychiatric Association, 1994). Conventional interpretations of severity of impairment by GAF scores are: Mild (61–70), Moderate (51–60) and Severe (41–50).

#### 2.2.2 Researchers

The study’s group of researchers consisted of a clinical psychologist and qualitative researcher (KD); a clinical psychologist (KB); and a psychiatrist and professor who was head of the pilot project (TW). KD, KB, and TW all clinically work part-time as psychotherapists, mainly with people diagnosed with personality disorders. Together, they have backgrounds in MBT, psychodynamic therapy, schema therapy, and MIT. All share an interest in psychotherapy research and personality disorders, and all three were involved in all aspects of the analysis.

#### 2.2.3 Interviews

The interview guide for the present follow-up interview was developed by TW as a semi-structured in-depth interview consisting of open-ended questions with potential follow-up questions. Questions pertaining to the patient’s subjective experiences of change were as follows: Do you feel different now from when you started treatment? If so, how? How significant do you think these changes (or lack of changes) are? Do you believe that anyone around you has noticed these or other changes? Have you any idea of what may have caused these changes? For instance, has something happened in your life that may have affected you? Do you think the treatment here has had an influence on you? In case of no change, do you think the treatment has had a negative impact or limited you in any way? Is there something that you missed in the treatment you received here? Do you think you have any personal qualities or resources that have been helpful?

The interviewers were instructed to pursue and explore any relevant themes that the individual patient touched upon during the interview. Most of the interviews were performed by two psychologists specializing in clinical psychology and one doctor specializing in psychiatry, none of whom were involved in the treatment. One additional interviewer had been the individual therapist of two patients in the pilot study but did not know the interviewees. The interviews were performed face to face, audiotaped, and later transcribed by three independent research assistants.

### 2.3 Qualitative methods

#### 2.3.1 Data analysis

As the aim of this study was to explore the participants’ experiences of change after participating in the treatment program, we chose to base our analysis within a hermeneutic-phenomenological epistemology and use reflexive thematic analysis within this context to search for themes or patterns of meaning within the data (Braun and Clarke, 2006, 2012, 2019). According to Braun and Clarke (2019), thematic analysis can be epistemologically positioned as being about meaning and meaning-making that is always situated, positioned, and context-bound and as viewing researcher subjectivity as a resource in knowledge production rather than a flaw in the analysis.

We inductively searched for themes that captured important aspects of the participants’ experiences of change. Thus, the first step of the data analysis consisted of a thorough reading and rereading of the interviews to begin engaging with the data and then searching for commonalities and variance across the main data set on a more descriptive level for initial coding. This led to the second step of the analysis, which concerned grouping the interviews according to whether their descriptions conveyed change, some change, no change, or a change for the worse. In the third step, we analyzed commonalities within these domains to generate initial themes (Braun and Clarke, 2006, 2019). This step concerned reading the sub-grouped data sets to search for patterns of meaning on a more interpretative level that could reflect the descriptions given by the participants.

In this context, the term “interpretative” means that researcher KD reread the grouped interviews several times to first develop a sense of what may be called “feeling states” conveyed by the various descriptions. These feeling states can be understood as the embodied, lived experience of the researcher reading the descriptions (Finlay, 2014) that creates felt impressions. Researcher KD utilized on these impressions when searching for patterns of meaning in a back-and-forth movement so as to let the more descriptive analysis inform the more experiential analysis and vice versa (Braun and Clarke, 2019). Our participants were often less articulate in their descriptions or less familiar with verbalizing inner experience, as is often the case for people struggling with AvPD (e.g., Dimaggio et al., 2015). Hence, this interpretative approach may inform the analysis of the rather “thin descriptions” provided in several of the interviews. Furthermore, the continuous rereading of the interviews across the grouped domains of change descriptions also allowed for the variance in descriptions in one category to inform and contrast the descriptions in another and, as such, add richness to the interpretations.

The research group then discussed and reflected together in several meetings to reach a consensus over the grouping of the interviews into the domains of experienced change and the initial themes. These themes were then further developed by rereading all the interviews to make sure that the themes coincided and worked with the data. This last step ensured that the themes were grounded in the participants’ descriptions and conveyed the meanings expressed.

#### 2.3.2 Reflexivity

To remain aware of our inductive stance toward the participants’ descriptions, we maintained constant focus on our fore understandings through holding discussions throughout the analysis. This involved continuous awareness of the researchers’ theoretical positions as well as the context of the interviews as part of a larger pilot to evaluate a specific treatment. We also reflexively discussed the felt, embodied meanings conveyed by the themes to remain aware of our own subjective experiences, colored by our individual contextual understanding. We sought this awareness to keep our analysis close to the participants’ meaning-making as theoretical interpretations and clinical experience were made salient.

### 2.4 Ethical considerations

This study was approved by the Regional Committee for Medical Research Ethics. All the participants provided informed signed consent to participate in the study, and all biographical data have been changed slightly to ensure the participants’ anonymity in the presentation of the qualitative findings.

## 3 Results

**Figure 1:**
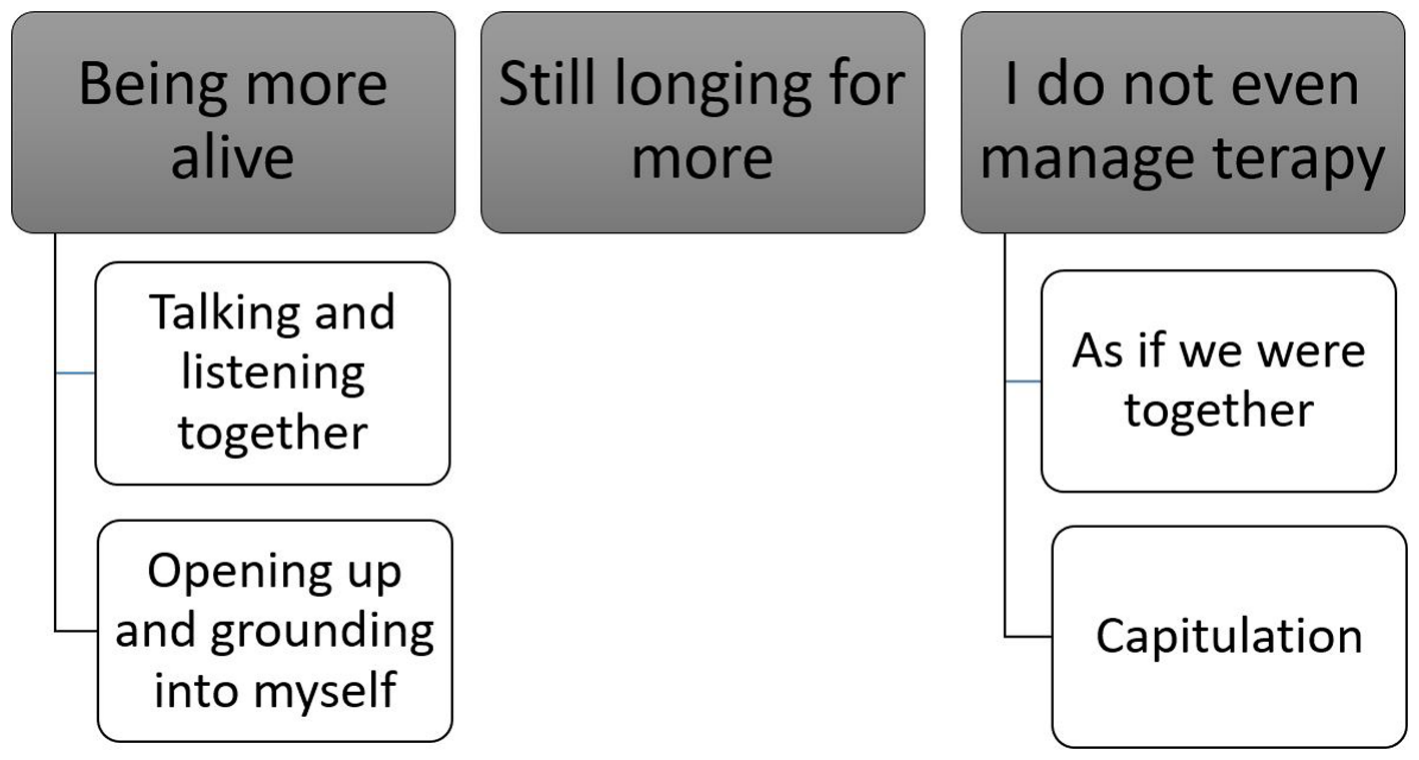
Overview of the main themes and subthemes.

The analysis of the participants’ descriptions supported three main themes, the first and third of which also included two subthemes found to capture the experiences conveyed (see Figure 1). The main themes (“being more alive,” “still longing for more,” and “I do not even manage therapy”) all represent various experiences of degrees and dynamics of change for better or for worse.

The representativeness of our findings is indicated by the grouping of the 18 interviews into categories of change: five interviews reflected “no change or change for the worse,” five interviews reflected “some change,” and eight interviews reflected “change for the better.”

The themes are described below with quotes from individual participants.

### 3.1 Being more alive

The first main theme, “being more alive,” was found to encompass the sense of vitality, shifting perspectives, and evolving agency that was conveyed by the participants who spoke of change for the better. Their symptoms and challenges were not gone but seemed reduced or manageable.

One participant shared the following: “It is like night and day… from everything being just black and dark and completely horrible, fearing the future not knowing what anything would be like, not relating to anyone, to life being so much easier. I see myself with new eyes. I am a lot less critical of myself, even though I am still quite critical. I can say about myself that I am quite good. I could never have said anything like that before.”

The participants’ descriptions conveyed development and movement, and the change seemed to be based within themselves. At the same time, the basis of the change came across as no longer disconnected and alienated from themselves or others but rather as though they were beginning to open up to new experiences. One participant talked about the direction of change as “just being a bit more alive,” which became the title of this main theme.

#### 3.1.1 Talking and listening together

Within this first main theme, the subtheme “talking and listening together” came to reflect a shift in what it had meant for the participants to talk together: to speak their own words out loud and to truly listen and take in the meaning of what the other person actually tried to communicate.

Some described the impact of listening to themselves talk out loud and expressing their thoughts to others. Not only was there a practice of talking to others, but it was as if the transference from thoughts to spoken words laid the groundwork for a change in what the words communicated, not just to others but also to themselves. As one participant put it, talking out loud could lead to a shift in perspective: “When I say things out loud, I suddenly see it from another angle.”

Furthermore, the effort and impact of entering a dialog where the participants take turns, moving back and forth between saying something to listening to replies and responding, became salient.

One participant shared the following: “Just to sit in a group, perhaps to be chosen to both share and to manage to listen. I think just that in itself was important. Just talk, sit there and talk about feelings and things to several people and just get confirmations and replies. The other way around, to manage to sit and listen. It was useful to keep focused on what the others said and give feedback.”

The participants particularly emphasized how being in the group therapy set these experiences of talking and listening in motion. There seemed to be a kind of revelation of discovering that the other group members had hidden inner lives, just as they themselves had. Not only did other participants describe thoughts and feelings similar to their own experiences, thus giving a sense of fellowship, but they also expressed ways of thinking and feeling that came across as surprising and strange to them. In others words, other group members came across as both similar and different to themselves, creating a sense of recognition and contrast in their relating to others.

In a sense, this seemed to involve an element of trust in getting to know each other: a trust in what was said, believing in it, then taking it in. This experience of trust seemed to put into motion the participants’ questioning of personal beliefs regarding how others think or feel about them, perhaps particularly the belief that everyone else would judge them as harshly as they judged themselves, and made them consider that they might not be as different from others as they had presumed.

One participant shared the following: “I have always believed that I was a freak, and then I see that there are actually normal people here that have just as many issues as me. And, I would never have seen it or thought that. You come to realize that you are not alone in feeling stuff. That others can feel and think completely weird things without you thinking that it is weird. Then I realized that I know what others think and it is likely not as bad as what I used to believe.”

It was as if the participants managed to let go of their own preconceived opinions and assumptions of others’ thoughts and feelings. There was a movement from being stuck “inside their own heads” toward somehow opening up from their inner world into a relational space or a common world of perspective exchanges, as captured by the next subtheme.

#### 3.1.2 Opening up and grounding into myself

The second subtheme of this main theme was related to a dynamic process of both “opening up” and “grounding into myself.” This theme conveys the beginning of a sense of calm, safety, surplus, and openness to new experiences. One participant said, “I have never felt relaxed ever. This is the most relaxed I have been so far in my life. I get time to do stuff I wish to do.”

Moreover, the participants described how they knew and felt their feelings had changed into something that was more like a part of them. Perhaps the avoidance had kept them stuck in a state or position of alienation from their own inner experiences. When daring to let go of the avoidance, their state changed into beginning a connection with their actual feelings and thoughts rather than just sensing all the bodily activation and arousal that made the urge to avoid so compelling. This emerging connection also appeared to enable a sense of release or becoming unstuck.

One participant shared the following: “Earlier, I did not know what I felt, or I knew, but I just blocked it, or hid some scary thoughts that I could not stand having. It took a lot of crying, the loosening of a lot of knots, but now I have less baggage and can concentrate on the present.”

When the present moment came into the participants’ awareness through paying attention to their experiences and feelings, leading them to getting to know themselves better, the participants’ comparison between the past and the present became enhanced. Furthermore, as the participants’ descriptions conveyed a distinction between the past and the present, a future seemingly emerged: one that was theirs and not just something to dread. It not only concerned the far future but also a sense that something new, perhaps experiences connected to their aspirations and desires, was possible. This could lead them in the direction they wanted and perhaps even expand their likes and desires.

One participant said, “I feel more content in general. Perhaps the biggest change is that I believe the future will be OK. I feel like I have many more possibilities. I can do more of what I want because I am not so afraid of many of the outcomes anymore. In addition, there are more things that I want.”

This sense of wanting more, the possibilities of new experiences, and the implied learning seemed connected to an awareness of having choices, an experience that was grounded in themselves. “I have taken other choices than I’ve done before and therefore have other experiences, and I have used that experience base to perhaps take choices that are better for me,” said one participant.

It was as if these movements of opening up and grounding into themselves were interrelated. The dynamics of opening up to take in more interaction and new perspectives seemed to ground the participants’ sense of being someone both similar and different to others. Likewise, getting to know themselves better seemed to give the participants an internal perspective from which to widen their scope and perhaps ground a sense of agency.

### 3.2 Still longing for more

The second main theme “still longing for more” reflects the experiences of the participants who described important changes but found their changes somewhat unsatisfactory. Some of these participants described feeling more stable and less anxious, having fewer panic attacks, and functioning better every day. Others spoke of how they felt a greater awareness or acceptance of themselves that alleviated their pain a little.

At the same time, a sense of longing for something more lingered. The participants’ awareness and acceptance seemed hesitant or questioning. Even though their functioning had improved, their inner experience seemed largely unchanged. “It is very nice that I function better now, but I am also afraid that this is how it will be for the rest of my life. Because it is going fine, but I do not think I can stand this for the rest of my life,” said one participant.

The participants described how meeting others with similar issues in a group setting was nice but that their overwhelming fears hindered a sense of feeling present and connected with the others. The result of this was that their experience in the group came to concern struggling with how to endure and cope, much in the same way they would do in other situations they feared.

One participant shared the following: “It was all right to meet others with similar problems, but I was just so nervous before every group session and extra nervous if I knew it was my turn to share something. Just to talk in front of people, to be in the center of attention is perhaps what I struggle with the most. So to be in a group was kind of like jumping into a fire.”

Thus, there was still a sense of standing on the sideline, mostly paying attention to their own inner experiences.

One dialog between a participant and the interviewer may shed light on what could be at the core of these participants’ longing:

“Participant: When I started, there was social anxiety, eh, hard to be around people. I do feel it sometimes in some special situations, but a lot less.

Interviewer: But that sense of loneliness, has that been reduced?

Participant: No, not really. So yes, that is most probably what has been the problem all the time.”

Therefore, although change and movement came across in these descriptions, the participants’ development appeared to be at a standstill or on the tipping point of daring to take the next step. Their aloneness and disconnection from others still seemed to be conceived as unshareable, remaining as enclosed parts of their inner reality. Moreover, their experience of positive change was present, but somehow, their change seemed to be perceived as something that had happened to them. It was as if they had not yet discovered that the source of change came from them relating to themselves and others differently and that they could be agents in their own lives.

### 3.3 I do not even manage therapy

The third and last main theme, “I do not even manage therapy,” reflects the descriptions of the participants who described no change or, rather, change for the worse. These participants described how they had not managed to succeed in therapy: they neither achieved what they thought others had expected from them nor what they themselves had hoped for. Some described being more aware of their problems, but that seemed to have made it even worse. It was as if they had lost their former ability to ward off their fear and suffering, feeling bereft of possibilities to change anything. One participant shared the following: “I feel more anxiety now than before, and I think it is because I have become more conscious of it. If I begin to feel uncomfortable, I get more aware of it and that makes it more uncomfortable, like I am in a state of emergency all the time.”

These participants had given up on trying and lost hope. They seemed to conclude that they had failed, were failures, and that not even a therapist could help them. A feeling of resentment came across in their descriptions, perhaps as expressions of a perceived underlying unfairness of their condition.

#### 3.3.1 As if we were together

Within this third main theme, the subtheme “as if we were together” reflects how these participants conveyed a sense of the absence of connection. It was as if they had stayed within their own minds, never entering the interpersonal space of the back-and-forth movements of increasing understanding between minds. Their focus in the group therapy seemed fully on planning their own presentations and striving toward being correct or flawless, resulting in little being said or shared. “I felt that I got to be quite focused on myself or on what I said and how I said it. If someone said something, I would sit and think about what I could have asked or said but be afraid that it would come out wrong or that it would not be a good question,” one participant said.

On the other hand, the responses they received after saying something did not convey the engagement or responses they had seemingly hoped for; thus, they resorted to almost acting like they were taking part in a play, unaware of how these disconnected acts influenced the interactions. “When I brought something forward, I felt like there was no interest or engagement. I just shared something when I felt like it had been too long since the last time I shared and I just had to share something,” one participant said.

The participants conveyed a sense of having wanted to open up or learn how to give and receive feedback, but they had not dared to and were given no help from the therapists. Furthermore, these descriptions gave off a sense of underlying tension from always having to wait while others talked or feeling as though others were more important. “I felt like I had no place in the group. That I was inferior and that everybody else’s problems and talking time counted more than me and mine,” one participant said. At the same time, it was as if the others were not relevant, like they had all been there together, alone within their own experiences.

#### 3.3.2 Capitulation

The second sub theme of the third main theme was “capitulation.” The participants had partaken in this therapy but had gotten nothing from it and that was it. It seemed like they were victims of their affliction, both giving up on themselves and others. As one participant expressed it, “I have perhaps become fatalistic. I guess this is what I can expect for the rest of my life.”

The whole experience lined up with similar attempts at getting help, exhausting the helpers, then concluding that they could not expect anyone else to endure them. As one participant put it, “When I think that I have exhausted yet another therapist, it seems like, wow, am I that strenuous?” The difference in this case was that specialized treatment could not help the participants, leading them to conclude that there had to be something particularly wrong with them, like a malfunction or a defect, or something they lacked. “Perhaps I just did not open up enough. Or perhaps I am just fucked. Perhaps I am just connected wrong in my head,” one participant said.

There was a sense of hopelessness among the participants, like there was nothing more to do besides accepting their fate and resigning themselves to it. Some said it was as if they had done something wrong during therapy, but they had no idea why and how they had failed. At the same time, the treatment also came across as being a nuisance, energy depleting, and an imposition. Nevertheless, when the treatment ended without any gains, the participants having been left alone and depressed, their desperation shone through yet again. “Am I to start over again? I cannot stand it. I cannot stand it,” one participant said. This state of giving up seemed unavoidable and unbearable to most of them.

## 4 Discussion

When putting these findings together, we may contrast the experiences of those who spoke of positive change with those who described beginning change and those who described change for the worse or no change. The participants who talked about positive development and change came across as being active participants in their own lives, taking a more knowing stance toward themselves and their wishes and desires while being aware of their challenges. They seemed to have established a foundational sense of trust in both themselves and others, as well as in their sense of mastery. Furthermore, if needed, there was the possibility of support from others. The participants who appeared to be in the early stages of more profound change, or talked of important changes and experiences like reduced anxiety, also described an awareness of still being in a state of distance from themselves and others, feeling alone, and in search of meaning. Finally, the participants who described being worse off or experiencing no change gave an impression of resignation and of being left alone and isolated with their frustration and despair. They conveyed the experience that no one could help them and there was no hope left for change to occur.

What comes across as a striking difference in these descriptions is the diversity in the sense of owning one’s own developmental movement toward something considered a change for the better or the worse. Moreover, the participants’ reports of change for the better seem related to interpersonal processes, mainly in the group therapy setting. This may suggest that variations in the experiences of connection and belonging versus the sense of loneliness are important aspects of our findings. We will now explore how our findings of how the participants’ experiences and meaning-making of change changed after attending a treatment program developed for AvPD can relate to theories of intersubjectivity and motivational systems, as well as to personal and relational agency.

Stern’s (2004) theory of intersubjectivity may shed some light on the participants’ contrasting experiences of belonging and loneliness. Stern (2004) considers our various forms of self-consciousness to evolve and take place within the intersubjective matrix. This matrix refers to the continuous dynamic dialog between minds in which minds are co-created: “two minds create intersubjectivity. But equally, intersubjectivity shapes the two minds” (Stern, 2004, p. 66). This is the realm in which we learn to know that others have minds of their own and that we can assume to know something about what they are feeling and thinking. Furthermore, we get to learn how the other is experiencing our experience of them and vice versa. In other words, the development of mentalizing abilities (Fonagy and Luyten, 2009) is assumed to take place within the intersubjective space Mentalizing or metacognitive abilities (the ability to understand mental states) are fundamental for inferring others’ as well as one’s own intentions, desires, goals, values, or motives so that we are able to know why we act as we do and interact with the motives of others (e.g., Stern, 2004). According to Stern (2004), intersubjectivity may also be viewed as a motivational system regulating the need for psychological belonging (Baumeister and Leary, 1995; Baumeister, 2012) as opposed to psychological aloneness. On one end of this dimension, we find fusion, enmeshment and disappearance of the self, and on the other end, pervasive loneliness. The comfort zone is found in between and is regulated within the interpersonal context available to the person (Stern, 2004). The need for belonging is viewed as separate from the attachment system but as equally fundamental. The systems facilitate each other and both motivate behavior (Stern, 2004; Lyons-Ruth, 2007; Cortina and Liotti, 2010). The attachment system may also be seen as regulating the needs and motives of proximity/security on the one hand and distance/exploration on the other (e.g., Stern, 2004).

The participants who described change for the better appeared to have both a sense of belongingness and uniqueness as well as to have established adequate attachment safety for exploration in their therapeutic contexts. Furthermore, the descriptions of the theme “opening up and grounding into myself” seem to capture aspects of how it may feel to connect intersubjectively and within this realm share one’s thoughts, emotions and personal stories; the experience of getting to know oneself through the minds of others implies feeling safe and understood, thus increasing a pull toward more interpersonal connections. In contrast, the experiences of this study’s participants who described some change, no change, or change for the worse could illustrate what may hinder connection, belonging, and the expansion of mentalizing abilities.

Theories of agency and the motivational system of social ranking could inform our understanding of what may have become barriers to the experience of belongingness, adequate attachment and intersubjective connection for the participants who experienced no change or change for the worse in therapy. Having a sense of being the source of our actions, that we are in control of our actions, and that they are linked to our intentions and motives are commonly referred to as personal agency (e.g., Moore, 2016). The way we perceive ourselves as being capable of agency is formed by our developmental experiences of how we were able to influence the conditions in our lives and determine our actions, as opposed to just becoming a product of what happens to us (Bandura, 2006; Huber et al., 2019a). A further expansion of our understanding of agency includes the concept of relational agency, which considers individuals to be interactants rather than singular actors (Burkitt, 2016; Gundersen, 2021). As interactants, we partake in interplays of both power imbalances between the actors and mutual interdependence and varying degrees of emotional relatedness that will influence our sense of agency within the various interpersonal and social relations (Gundersen, 2021). Power imbalances are considered an inherent element of another important social motive, namely, the formation of social ranks and competition for resources through behavioral strategies related to dominance and submission (e.g., Liotti and Gilbert, 2011; Dimaggio et al., 2015). Blay et al. (2021) suggest that people who have high social anxiety, a condition often associated with AvPD (e.g., Eikenaes et al., 2013; Lampe and Malhi, 2018), may be conflicted between their desire to gain ranking and status and the urge to avoid defeat and humiliation. At the same time, the desire for interpersonal connection may be in conflict with the desire to avoid rejection. Furthermore, a preoccupation within one area of social motivation, e.g., social rank, in one context may come at the expense of another, e.g., belonging, in the same context.

So, perhaps the participants who fared worse experienced their relational agency as poor: How could they be in control of what happened to them in an interpersonal space driven by both their need to position themselves within the social rank system and their need for belonging when all their previous experiences had likely taught them that control is out of their reach? Many people diagnosed with AvPD have a history of neglect and trauma, bullying, a growing relational and emotional distance to others, and a fear of abandonment (Rettew et al., 2003; Eikenaes et al., 2015; Hageman et al., 2015; Sorensen et al., 2020). Thus, their perception of having to adapt to others, fearing the intentions of others, and feeling excluded in the social or intimate world may likely reduce or limit their sense of both personal and relational agency (Sorensen et al., 2019a). Kunst et al. (2020) found early maladaptive interpersonal schemas relating to the domain of disconnection and rejection to be associated with AvPD. Their strategies of resorting to interpersonal submission and withdrawal make sense in the likely context of their developmental experiences (Horowitz et al., 2006; Huber et al., 2019b). Since approach strategies to improve rank or position, as well as to connect with others, could seem like a certain road to failure, avoidance strategies are implemented to reduce the likelihood of humiliation and rejection at the cost of their sense of intersubjective connection and belongingness. Retreating into avoidance strategies will naturally reduce the possibility of connection and cooperation that lay the ground for intersubjective experiences of getting to know each other’s minds through dynamic attuned dialogs (Sorensen et al., 2019a). The subjective experiences captured in the “as if talking together” theme in our findings indicate that the participants take a position of never revealing much or just pretending to interact in order to maintain a distance from others. Then again, their discomfort of feeling isolated that results from this distancing strategy could entail an inherent push toward more connection, which again increases the fear of humiliation and rejection in the social rank system. Fear of the consequences of becoming salient to others and to oneself and that of what will happen if all that has been avoided is brought forward to attention, like the mistrust of the responses from others, the strong perception of inferiority, and the fantasized rejection, may be intense. One of the participants indeed compared being in the group to “jumping into a fire.” Thus, the resignation expressed by these participants may reflect the feeling of being stuck stemming from this conflicting position, perhaps intensified by an ongoing pull from others to connect. The only way to execute any agentic influence may be through the paradoxical act of giving up on positive change.

The participants who described change for the better may be less conflicted between or ambivalent toward their motivations for belongingness and social rank. We may speculate that their drive toward connection became stronger than their fear of humiliation, resulting in them opening up to new learning experiences and increasing their sense of agency. These participants may have discovered that they can influence the way they perceive others and the way others perceive them. They opened up to reconnect with themselves and gained more awareness of their own affects. From this comes direction and intentionality and, thus, also the possibility of a future that they themselves may influence. Within this understanding, it also becomes salient that improvement and change do not exclude having challenges or difficulties in life; rather, one develops a sense of being able to manage adversity.

The group of participants who found themselves somewhere between these two positions of change may also be interpreted through the theories of intersubjectivity, personal and relational agency, and social motivations. The participants who were “still longing for more” came across as still feeling alone and being insecure and hesitant about daring to cross the barrier of fear into the intersubjective matrix where they would become known by others. Descriptions of important improvements, like reduced social anxiety, may indicate that they were in the initial stage of testing out new social interaction behaviors with reduced fear of humiliation but still not feeling secure enough to venture into intersubjectivity that could provide a sufficient sense of connection. It might be that the predefined time period of the specialized treatment program was simply too short for these participants to become secure enough to try out new intersubjective behaviors.

On the other hand, there could be other aspects of the participants’ everyday life that were of greater importance than therapy in fostering or hindering their personal development: their economic situation, interpersonal relations, and work or leisure activities or the lack of either.

Therapy of all forms involves some form of exposure to one’s fears, and therapy for AvPD is no exception. Yet, the sought after ideal for group therapy is to create an egalitarian, cooperative, and safe atmosphere of trust and sharing that allows for openness, exploration, and new learning experiences. The interpretation of our findings from the perspective of intersubjectivity, motivational systems, and agency may guide therapists in creating therapeutic relationships that regulate fear and support development. Being attentive to and exploring what intentions lie behind behaviors and strategies may be of help in a process that allows for the dynamics of intersubjectivity to evolve. While many therapists are well trained to focus on attachment and the emotional bond between the patient and the therapist or group members, other social motives, like social rank, have in general received less attention. However, internal conflicts between social motivations may become apparent in therapy (Dimaggio et al., 2015). For instance, expectations of connection can be associated with threats, e.g., when a group member or therapist initiates the process of getting to know someone when that person thinks becoming known will lead to humiliation and ridicule. Thus, depending on what social motivation guides a person’s perception of what is taking place in an interaction, the intentions of others might be misinterpreted. Expressions of empathic understanding could be perceived as belittling, and the person is likely to ward off the empathy and withdraw from the interaction.

Learning about our various social motivations, interpersonal schemas and agency may contribute to normalize the internal conflicts that patients with AvPD experience in social relations. Thus, for therapists working with patients with AvPD, attending and attuning to the patient’s primary need and motivation at any given moment in time could better guide interventions so that the fear of humiliation and rejection might be reduced, thereby facilitating a sense of connection, belonging, security, and trust. For patients with AvPD, this might improve the sense of having a therapeutic alliance with the therapist. From this perspective, the motivations set in motion could potentially be supportive of each other rather than conflicting. For instance, if a person’s social rank motive is validated and normalized, and their presumed low social rank becomes explored and challenged, openness and cooperation may lessen the negative impact of their motive and restore their sense of belongingness. Furthermore, their interpersonal connection may improve their social standing in itself as new social learning experiences can challenge maladaptive interpersonal schemas, expand one’s mentalizing abilities, and improve one’s sense of agency.

## 5 Limitations

The aim of this qualitative inquiry was to further our understanding of the subjective meaning of change in a specific therapeutic context adapted to patients diagnosed with AvPD. The participants’ accounts of their experiences are subjective and retrospective and are not to be considered as objective or generalizable. Moreover, the time for the interviews as well as the participants’ life experiences during the period after the treatment ended may have influenced their recollection and sense-making. Furthermore, the findings cannot be considered specific to AvPD patients, patients attributed specifically to MBT or MIT, or the qualities of the therapists. On the other hand, the participants were recruited in an outpatient hospital setting and could thus be representative of patients with moderate to severe impairments, as many of them had several previous treatment experiences. Thus, our findings may inspire future research investigating change processes, agency, and adaptions of therapeutic interventions for patients diagnosed with AvPD.

## 6 Conclusion

The present findings inform our understanding of the subjective experiences and sense-making of change of people diagnosed with AvPD after participating in a treatment program. Comparing the experiences of those who experienced positive change, some change, no change, and change for the worse pointed us toward paying attention to intersubjectivity and the interpersonal dynamics of social motivations and sense of agency. The flux of these dynamics may foster or hinder intersubjective learning and development. Therapeutic awareness of the various social motivations of patients may be important for facilitating a sense of trust and intersubjective connection in the interpersonal context of therapy. When the sense of connection and agency grows, the potential for change can be nurtured and opened up for further positive learning and development.

## Data availability statement

The datasets used for this study are not publicly available to ensure the participants’ privacy and anonymity. Requests to access the datasets should be directed to TW, UXTHWI@ous-hf.no.

## Ethics statement

The studies involving humans were approved by the Regional Committee for Medical Research Ethics. The studies were conducted in accordance with the local legislation and institutional requirements. The participants provided their written informed consent to participate in this study.

## Author contributions

KD: Formal analysis, Methodology, Writing – original draft, Writing – review & editing. KB: Formal analysis, Writing – review & editing, Investigation. TW: Formal analysis, Project administration, Writing – review & editing, Investigation, Data curation, Methodology.
